# Dual-Specificity Phosphatase Regulation in Neurons and Glial Cells

**DOI:** 10.3390/ijms20081999

**Published:** 2019-04-23

**Authors:** Raquel Pérez-Sen, María José Queipo, Juan Carlos Gil-Redondo, Felipe Ortega, Rosa Gómez-Villafuertes, María Teresa Miras-Portugal, Esmerilda G. Delicado

**Affiliations:** Departamento de Bioquímica y Biología Molecular, Facultad de Veterinaria, Instituto Universitario de Investigación en Neuroquímica (IUIN), Instituto de Investigación Sanitaria del Hospital Clínico San Carlos (IdiSSC), Universidad Complutense Madrid, 28040 Madrid, Spain; mqueipo@ucm.es (M.J.Q.); jugil@ucm.es (J.C.G.-R.); fortegao@ucm.es (F.O.); marosa@ucm.es (R.G.-V.); mtmiras@ucm.es (M.T.M.-P.)

**Keywords:** dual-specificity phosphatases, MAP kinases, nucleotide receptors, P2X7, P2Y_13_, BDNF, cannabinoids, granule neurons, astrocytes

## Abstract

Dual-specificity protein phosphatases comprise a protein phosphatase subfamily with selectivity towards mitogen-activated protein (MAP) kinases, also named MKPs, or mitogen-activated protein kinase (MAPK) phosphatases. As powerful regulators of the intensity and duration of MAPK signaling, a relevant role is envisioned for dual-specificity protein phosphatases (DUSPs) in the regulation of biological processes in the nervous system, such as differentiation, synaptic plasticity, and survival. Important neural mediators include nerve growth factor (NGF) and brain-derived neurotrophic factor (BDNF) that contribute to *DUSP* transcriptional induction and post-translational mechanisms of DUSP protein stabilization to maintain neuronal survival and differentiation. Potent *DUSP* gene inducers also include cannabinoids, which preserve DUSP activity in inflammatory conditions. Additionally, nucleotides activating P2X7 and P2Y_13_ nucleotide receptors behave as novel players in the regulation of DUSP function. They increase cell survival in stressful conditions, regulating DUSP protein turnover and inducing *DUSP* gene expression. In general terms, in the context of neural cells exposed to damaging conditions, the recovery of DUSP activity is neuroprotective and counteracts pro-apoptotic over-activation of p38 and JNK. In addition, remarkable changes in DUSP function take place during the onset of neuropathologies. The restoration of proper DUSP levels and recovery of MAPK homeostasis underlie the therapeutic effect, indicating that DUSPs can be relevant targets for brain diseases.

## 1. Introduction

Dual-specificity phosphatases (DUSPs) comprise a family of protein tyrosine phosphatases (PTPs) with wide substrate selectivity and are known as powerful regulators of biological processes. They are Cys-based phosphatases and exhibit dual activity towards Ser/Thr and Tyr residues in their catalytic substrates. Among the DUSP family, MKPs or mitogen-activated protein kinase (MAPK) phosphatases form a small group of 10–12 phosphatases that selectively dephosphorylate MAP kinases. Although MAPK signaling can be modulated at different levels, MKPs exert tight control, deactivating MAPKs with a 10–100 stronger potency than the upstream kinases. Therefore, they are emerging as the most potent regulators of the duration and magnitude of MAPK signaling and function [1,2,3]. While DUSPs encompass a greater family of phosphatases, this term will be used in the present review to refer to the MAPK-specific group. 

Many of the physiological implications of DUSPs, including their substrate selectivity, modes of regulation, and function, have been elucidated through the genetic expression or deletion of a specific DUSP subtype in cell lines and heterologous expression systems. However, the way in which they are regulated by extracellular messengers, trophic factors, or toxic insults still remains poorly understood. The best-known extracellular mediators regulating DUSPs are trophic factors. Indeed, DUSPs form part of the feedback regulation of MAPK signaling during proliferative responses induced by mitogens. Initially, mitogens activate MAP kinases, which in turn promote *DUSP* expression and/or their stabilization to terminate MAPK actions. In this context, the participation of DUSPs in the regulation of proliferative processes is well-documented and DUSPs have become a major focus of cancer research. In agreement, significant changes of *DUSP* expression levels in tumor cells support their role as important markers of the stage, progression, and prognosis of certain types of cancer. Some DUSPs evolve with a gain of function and behave as tumor suppressors. On the other hand, the loss of *DUSP* expression is usually associated with sustained proliferative responses and chemo-resistance in other cancer types, as seen with *DUSP6* hypermethylation in pancreatic cancer [4,5,6].

Data from the knock-out mice models have also been important to identify the important role that DUSPs have in the regulation of immune responses and metabolic homeostasis. With regard to this, genetic knock-out models of DUSP1 and *DUSP10* present a pro-inflammatory phenotype, characterized by an increase in the production and release of pro-inflammatory cytokines. In contrast to this, DUSP2 seems to function as a positive regulator of inflammatory responses [7,8,9]. 

From the perspective of the nervous system, however, little is known about the function of DUSP proteins and how extracellular messengers can regulate them. In fact, most studies concerning intracellular cascades activated in neural cells by neurotransmitters or neurotrophic factors have classically focused on signaling kinases and have always eluded the deactivation mechanisms. The present study covers the different modes of regulation of DUSP proteins described in neuronal and glial cell populations, especially for DUSP1 and DUSP6, which appear to be the most studied DUSPs in neural cells. Special attention will be paid to the role of growth factors and neurotrophins, such as the brain-derived neurotrophic factor (BDNF) and nerve growth factor (NGF), which provide relevant examples of the complex regulation of *DUSP* expression through different intracellular mechanisms in neural cells. In addition to neurotrophins, endogenous cannabinoids, well-known messengers in the nervous system and key regulators of synaptic activity, also have the ability to modulate DUSP function at different levels with relevant importance in pathological contexts. It is interesting to point out that extracellular nucleotides, ATP, and analogues become novel players of DUSP function in neural models. They activate nucleotide receptors and share the intracellular coupling to MAPK mechanisms of activation and deactivation with trophic factors [10,11]. Furthermore, DUSP regulation by nucleotides is essential for cell maintenance and survival [12,13,14,15]. The physiological meaning of DUSP regulation in neural systems is further analyzed concerning processes of neuroprotection against different cytotoxic insults and in pathological situations, neurodegenerative diseases, neurological disorders, and brain tumors. From this point of view, DUSPs represent new targets for extracellular mediators whose modulation seems to be essential for the maintenance and homeostasis of the nervous system.

## 2. Overview of Regulatory Mechanisms of DUSP Activity

The DUSP family has been classified into three groups based on sequence homology, subcellular localization, and substrate specificity. All of them share a common structure consisting of an N-terminal regulatory domain and a C-terminal catalytic domain. Some types also contain specific motifs responsible for specific subcellular location [1]. The first group includes four inducible nuclear phosphatases. DUSP1/MKP-1 was the first MKP to be cloned and characterized and has been extensively studied in terms of regulation and function in different cellular systems. DUSP1 is a nuclear and inducible phosphatase whose specificity can vary from extracellular signal-regulated protein kinase (ERK) to the stress kinases, p38 and c-Jun N-terminal kinase (JNK), depending on the cellular model and the cellular context. Other members of this subgroup are DUSP2/PAC-1, DUSP4/MKP-2, and DUSP5. The second group includes three cytoplasmic ERK-specific DUSPs: DUSP6/MKP-3, DUSP7/MKP-X, and DUSP9/MKP-4. DUSP6 is the most representative ERK-directed phosphatase that can be slowly induced and primarily acts in the cytoplasmic compartment. The third group includes DUSP8/hVH-5, DUSP10/MKP-5, and DUSP16/MKP-7, which specifically dephosphorylate stress kinases p38 and JNK and can be either nuclear or cytoplasmic. Different DUSP subtypes are co-expressed in the same cell, but they do not exhibit redundant activities and usually function towards different MAPK substrates and in different subcellular compartments. 

The regulation of MAPK signaling by DUSPs is far more complex than initially expected, taking into account the different levels at which these phosphatases can be modulated. Modulation by transcriptional, post-transcriptional, post-translational, and epigenetic mechanisms has been described and is covered in excellent reviews [1,3,6,16,17,18]. 

The expression levels and regulation of DUSPs are cell-type- and context-specific and differ depending on their constitutive or inducible nature. The inducible protein phosphatases, such as DUSP1, behave as immediate early genes and are rapidly induced in response to growth factors, cytokines, and stressful stimuli. Nevertheless, even constitutive phosphatases, such as DUSP6, can be transcriptionally induced with delayed kinetics by several extracellular mediators like mitogens and growth factors, which activate MAPK signaling. Therefore, both MAPKs and DUSPs act in a coordinated fashion through negative feedback loops to regulate the proper duration and magnitude of MAPK signaling. The best-known model is provided by DUSP6, whose transcriptional expression is strongly dependent on ERK activity, through the activation of the E twenty-six (Ets) family of transcription factors [19,20]. However, cross-talk regulation is also possible and DUSPs can dephosphorylate different MAPK substrates to those responsible for their expression. In fibroblasts, the activation of p38 following UV radiation contributes to inducing *DUSP1* transcription, which in turn functions to dephosphorylate and attenuate JNK apoptotic signaling [21,22]. A wide variety of transcription factor downstream MAPKs can activate *DUSP* transcription, the most well-characterized of which are AP-1, SP-1, NFKB, and β-catenin [17]. Concerning *DUSP1* gene induction, glucocorticoid response elements (GRE) are present in the *DUSP1* promoter and recruit specific cofactors to facilitate *DUSP1* transcription. *DUSP1* transcriptional regulation lies behind the anti-inflammatory effect of glucocorticoids, such as dexamethasone [23,24]. Additionally, epigenetic modifications also take place through chromatin remodeling to facilitate *DUSP* transcriptional induction. The enhanced acetylation and phosphorylation of histone H3 during the arrival of stressful stimuli precede *DUSP1* transcriptional activation in mouse fibroblasts [25]. 

In addition, different modes of regulation can converge with transcriptional induction to finely modulate *DUSP* expression levels, such as mRNA stabilization. During apoptotic signaling in HeLa cells, MAPK activation plays a double role by favoring the transcription of *DUSP1* and, at the same time, increasing the translation of several genes that help to stabilize *DUSP1* mRNA and increase its half-life, such as the RNA binding protein HuR. These mechanisms of *DUSP1* up-regulation help to counteract JNK apoptotic signaling [26,27]. *DUSP6* mRNA stabilization also forms part of the mechanism by which *DUSP6* expression levels increase during hypoxia. This regulation depends on mitogen extracellular-activated kinase (MEK)-ERK and hypoxia-inducible factor (HIF)-I activity. Two novel factors have been identified as regulating DUSP6 stability (Tristetraprolin (TTP) and PUM2 in HEK293 cells [4].

Besides transcriptional induction, several other modes of regulation take place upon MAPK substrate binding through the kinase interacting motif (KIM) domain. Conformational changes near the catalytic site are behind the increase in DUSP activity [6,28,29,30]. In addition, like other PTPs, DUSP proteins are sensitive to oxidative stress. The cysteine residues located in the catalytic cleft are susceptible to reversible oxidation by reactive oxygen species (ROS), which renders phosphatases catalytically inactive [31,32,33]. The oxidized sulfonic acid intermediate forms disulphide bonds with nearby cysteines to prevent the irreversible formation of higher oxidation states. With respect to DUSP6, multiple cysteine residues in a distal domain function to bridge the active site and prevent it from excessive oxidation and irreversible inactivation [33,34].

**Figure 1:**
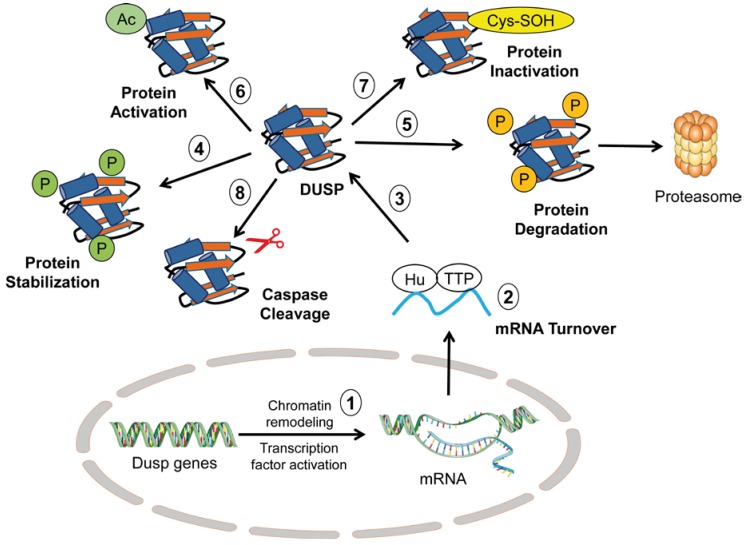
Regulation of dual-specificity protein phosphatases (DUSP) protein levels and activity. DUSPs can be regulated at different levels by their own substrates, mitogen-activated protein (MAP) kinases at (**1**) transcriptional, (**2**) post-transcriptional, and (**3**) post-translational levels by several mechanisms. The latter involve (**4**,**5**) phosphorylation, (**6**) acetylation, (**7**) cysteine oxidation, and (**8**) caspase–mediated cleavage. These modifications can positively or negatively affect DUSP activities, as well as act as a shuttle to subcellular compartmentalization.

Regarding post-translational mechanisms, these mainly involve processes of DUSP phosphorylation triggered by MAPKs. The final effect can vary, from enhancing DUSP turnover to stabilizing the DUSP protein and increasing its half-life [17]. Several residues have been identified that, upon phosphorylation, target DUSP proteins for proteasomal degradation. The best-known example is DUSP1, which is phosphorylated in Ser-323 and Ser-329 residues in the catalytic domain by ERK to allow the interaction with specific ubiquitin ligase, resulting in DUSP degradation [35,36]. However, in adjacent residues at the carboxyl terminal sequence, ERK-mediated phosphorylation of Ser-359 and Ser-364 exerts the opposite effect, augmenting DUSP1 protein stability. The same mechanism occurs in corresponding residues of DUSP4 [35,37]. Far from the catalytic domain, Ser-174 and Ser-197 in the amino terminus of DUSP6 are also susceptible to ERK-mediated phosphorylation that decreases its half-life [38,39]. Besides MAPKs, other upstream kinases gain importance in DUSP regulation, as happens with mTOR-dependent DUSP6 phosphorylation in Ser-159 that also leads to protein degradation [40]. Moreover, casein kinase-2α (CK2α) and protein kinase A (PKA) can differently affect both DUSP catalytic activity and MAPK binding and recognition [41,42]. Besides phosphorylation, other mechanisms of regulation have been described, such as DUSP1 acetylation. DUSP1 is acetylated in response to inflammatory stimuli and the activation of Toll-like receptor signaling. Modification at the Lys-57 residue in the KIM domain by p300 increases its association with p38 and its catalytic activity to stop inflammatory signaling [43]. Figure 1 summarizes the regulatory mechanisms of DUSP activity previously described. 

Finally, a novel regulatory mechanism described for DUSP6 connects it with pro-apoptotic signaling. The inter-domain linker region of DUSP6 is located between the N-terminal MAPK-binding domain and the C-terminal catalytic domain and is the target of the apoptosis executioner caspase-3. In response to apoptotic stimuli, active caspase-3 cleaves DUSP6 at the level of the linker region and renders several truncated fragments with different catalytic activities that regulate ERK subcellular location and activity [44]. All these data together do not exclude the possibility that further new residues and mechanisms not yet identified could be relevant to regulating the kinetics and turnover of different DUSP types (Figure 1). 

The complex regulatory network points out the importance of DUSPs that do not merely function to switch off intracellular cascades, but emerge as fine-tuners of MAPK signaling. Nevertheless, their role as MAPK regulators is not exclusive for DUSPs. It is necessary to take into account that constitutive Ser/Thr phosphatases also contribute to regulating the intensity of MAPK signaling, acting at multiple levels in upstream kinases. However, the lack of specific genetic tools and specific inhibitors makes it difficult to evaluate their real contribution. Besides, the control exerted by DUSPs at specific subcellular compartments and over specific MAPK substrates makes them better-quality regulators, allowing dynamic adaptations to different conditions and cellular contexts [2]. It is interesting to note that many of these regulatory mechanisms also take place in neural cells, which can have a special meaning in different physiological and pathological contexts.

## 3. DUSP in Neuronal Differentiation and Nervous System Development

Consistent with the role of MAPKs in proliferation and differentiation processes, MAPK deactivation is crucial to ensuring precise levels of neurite and axonal arborization. One of the most used in vitro models for neuronal differentiation studies has been rat PC12 pheochromocytoma cells. PC12 cells acquire a neuronal phenotype after treatment with NGF. In fact, NGF was the first neurotrophic factor reported to regulate *DUSP* expression in PC12 cells and embryonic sympathetic neurons. NGF induces *DUSP1*, *DUSP4*, and *DUSP6* gene expression during the first hours of incubation. *DUSP1* and *DUSP4* mRNAs appear during the first hour of incubation with neurotrophin, according to the inducible nature of these phosphatases, whereas *DUSP6* that appears later on, after three hours of incubation [45,46]. In previous studies, it has been demonstrated that NGF induces the sustained activation and nuclear translocation of ERK1/2 and cytosolic MAP kinase dephosphorylation and inactivation, observed after only 3 h of NGF treatment. The coincidence of the temporal window suggests that ERK could be the MAPK responsible for *DUSP6* induction. In addition, increases in the expression of DUSP1 and DUSP6 phosphatases are also detected during long-term incubation with NGF (one to four days), agreeing with the long-term actions of MAPKs in the context of NGF-induced PC12 cell differentiation [47]. Watezig and Herdergen proved that ERK1/2 and JNKs, but not p38, are crucially involved in the long-term differentiation of PC12 cells; JNKs alone are responsible for the phosphorylation of c-Jun and the expression of *DUSP1*. Supporting the role of DUSP in neuronal differentiation, *DUSP1* is also induced by NGF in embryonic dorsal root ganglion neurons [48]. In the P19 stem cell line differentiated with retinoic acid, *DUSP6* expression takes place earlier and more strongly, while the *DUSP1* transcript peaks are transient and slightly decrease during the course of differentiation. Only after the levels of DUSP6 and pERK decrease in the cytoplasmic fraction is DUSP1, together with pERK, detected and accumulated in the nucleus [49]. 

Continuing with neurotrophins, the elegant work of Jeanneteau and co-workers shows that the BDNF neurotrophin regulates the expression of *DUSP1* during the differentiation of mouse cortical neurons. BDNF provides different regulatory mechanisms of DUSP1, both at the level of gene transcription and protein stabilization. In primary cultures of mouse cortical neurons, BDNF acts as a strong inducer of the *DUSP1* mRNA. The transcriptional expression of the *DUSP1* gene is clearly dependent on ERK activation. Although *DUSP1* overexpression reveals broad substrate selectivity towards the three types of MAPKs, JNK appears to be the preferred DUSP1 substrate responsible for the effect of BDNF in axonal outgrowth and branching [50]. Using different conditional expression systems, it was possible to determine that BDNF also contributes to DUSP1 protein stabilization and sustains the increase in DUSP1 levels during BDNF stimulation. Additionally, the DUSP1 protein is lost upon the expression of some mutants of ERK-targeted residues, indicating that BDNF-induced DUSP1 phosphorylation at Ser-359 and Ser-364 might be responsible for protein stabilization and the increase in DUSP1 half-life [35,50]. The sustained DUSP1 activity is necessary to achieve adequate levels in phosphorylation of DUSP1 substrates that regulate cytoskeletal dynamics and result in permanent changes in axonal branching during differentiation. In this model, *DUSP1* primarily acts on JNK-phosphorylated targets that destabilize microtubules and allow cytoskeletal remodeling. The effect of BDNF in promoting axonal branching is dependent on the expression of *DUSP1* and is lost in cortical neurons obtained from *DUSP1*^−/−^ knock-out mice [50]. Similar to cortical neurons, DUSP1 expression is also important in the development of the dopaminergic system during the critical period of striatal axogenesis. When overexpressed, DUSP1 increases the neuronal complexity of TH+ dopaminergic neurons, as noted by the increase in neurite length and neuronal branching. In this case, it is p38 that is responsible for *DUSP1* expression and mediates its effect on differentiation [51].

DUSP6 emerges as a critical regulator of fibroblast growth factor (FGF) downstream actions on cell proliferation and patterning during the development of zebrafish, chick, and mouse embryos [52]. In these systems, DUSP6 forms part of a signaling loop of ERK-activation in the FGF-activated pathway. First, DUSP6 localizes in specific sites or domains of FGF activity in developing embryos [53,54,55]. Second, FGF signaling is responsible for *DUSP6* induction, mainly dependent on the ERK-MAPK pathway, although the PI3K/Akt axis also seems to contribute to *DUSP6* expression in the mouse neural tube [53,54,56,57]. The impact of DUSP6 as a brake of FGF-mediated signaling is based on aberrant proliferation and alterations in neuronal cytoarchitecture observed in developing of chick limb and mouse neural plate under conditions of *DUSP6* overexpression [53,55]. The specificity of DUSP6 actions on ERK activity in embryos has been confirmed by silencing studies and by the use of a BCI DUSP1/6 specific inhibitor, which prolongs increases in pERK levels [54,58,59]. 

All these findings indicate that DUSPs also behave as critical players during neural cell development and stem cell differentiation. Particularly, *DUSP1* and *DUSP6* are induced in a spatio-temporal manner to limit the extent of proliferative MAPK signaling, which is necessary to refine neural cell populations, restrict over-proliferation, and ensure precise levels of synaptic connectivity. DUSP6 seems to be more associated with proliferative mediators, such as FGF. By contrast, a *DUSP1* expression peak occurs at later stages of embryo development, when neuronal networks are being refined in the prefrontal cortex, hippocampus, and striatum [50]. *DUSP1* expression correlates with synaptic activity and is required for proper dendritic growth and axonal arborization, as *DUSP1* gene deletion abrogated these processes. Furthermore, the overexpression of this protein phosphatase is associated with abnormal cytoarchitecture of newborn neurons [50,60].

## 4. DUSP in Neuroprotection; Role of Neurotrophins and Nucleotides

Neuroprotection covers the intracellular mechanisms triggered to recover cell viability in response to brain injury and neurodegenerative events. Common hallmarks of neuronal death include excitotoxicity and oxidative and genotoxic stress. In all these conditions, alterations in MAPK signaling are behind cell death elicited by different kinds of cytotoxic insults and apoptotic stimuli. The general rule points to sustained increases at the level of MAPK activation over time. This is particularly evident for stress kinases, p38 and JNK, but also for ERK proteins. Whether the increase in MAPK activation constitutes a defense mechanism or anticipates cell death is dependent on the kinetics and duration of MAPK activation and their action on cytosolic or nuclear downstream targets. For instance, prolonged MAPK activation over critical cytosolic substrates can induce cytoskeletal rearrangements that are toxic to the cell. On the other hand, the activation of transcriptional nuclear targets can be either a protective mechanism, when increasing the expression of survival genes, or detrimental, through the induction of the pro-apoptotic program. In terms of the balance between survival and apoptosis, fine-tuning of spatio-temporal dynamics of MAPK activation will determine the final outcome.

A great deal of evidence points towards a failure in MAPK deactivation mechanisms as major contributors of prolonged MAPK signaling. Among different protein phosphatases, dual-specificity phosphatases become dysregulated in damaging conditions and brain pathologies. Different mechanisms impair DUSP catalytic activity and can concur to elicit cell death in neural cells, such as increased turnover and down-regulation of DUSP proteins, transcriptional inhibition, and oxidative inactivation.

### 4.1. DUSP Regulation in Excitotoxicity and Oxidative Stress

Oxidative stress is common to several apoptotic insults, being majorly responsible for cell death in brain injury, neurotoxic conditions, ischemic insults, and neurodegenerative events. A direct link exists between oxidative stress and the ERK signaling alterations [2]. The prolonged ERK signaling during these conditions is mainly attributed to dysregulation of the PP2A Ser/Thr phosphatase. But also, ERK-directed dual-specificity phosphatases contribute to the ERK over-activation, as they become catalytically inactivated by the oxidation of key residues. Several studies revealed that excitotoxicity induced by high extracellular glutamate concentrations causes oxidative damage to DUSP phosphatases and cell death. Primary hippocampal and cortical neurons are particularly vulnerable to glutamate-induced oxidative toxicity [61,62,63,64]. Loss of cell survival correlates with sustained ERK activation and its persistent translocation to the nucleus [65]. Although the initial peak of ERK activation functions as a first line defense mechanism, the delayed increase in ERK activity is harmful [63]. The induction of cell death by several phosphatase inhibitors points to the failure of ERK-directed phosphatases in toxicity. Among them, the PP2A serine-threonine phosphatase seems to play a major role, but also the inactivation of tyrosine and dual-specificity phosphatases contributes to maintaining of the ERK activity in later stages of glutamate-induced excitotoxicity [65]. The mechanism underlying phosphatase inactivation might involve the reversible oxidation of key cysteine thiols in the DUSP catalytic domain during oxidant conditions, as DUSP6 catalytic activity recovers by the use of a DTT reducing agent [65]. In addition, degradation of the DUSP1 protein through the protein kinase C (PKC)δ-dependent pathway occurs during glutamate treatment and is responsible for augmented ERK signaling in hippocampal and cortical neurons. The use of proteasome inhibitors or PKCδ silencing restores DUSP1 protein levels and recovers cell survival [62]. Additionally, the overexpression of *DUSP1* and *DUSP6* by different genetic approaches re-establishes basal pERK levels and cell survival after glutamate-induced damage in hippocampal neurons [62,64]. This is in agreement with the process described in non-neural models, such as rat mesangial cells, in which *DUSP1* expression exerts a prominent cytoprotective role in oxidative conditions of exposure to hydrogen peroxide (H_2_O_2_) [66]. 

*DUSP6* becomes down-regulated in oxidative conditions after H_2_O_2_ treatment and promotes persistent ERK activation in SH-SY5Y human female neuroblastoma cells. To the same extent as H_2_O_2_, treatment with a DUSP1/6 inhibitor, BCI, at critical concentrations, also induces cell death. This effect is accompanied by a robust increase in ERK phosphorylation and a concomitant decline of DUSP6 levels. Again, the recovery of *DUSP6* expression by 3α-androstanediol prevents toxicity induced by H_2_O_2_ challenge and BCI [67]. 

**Figure 2:**
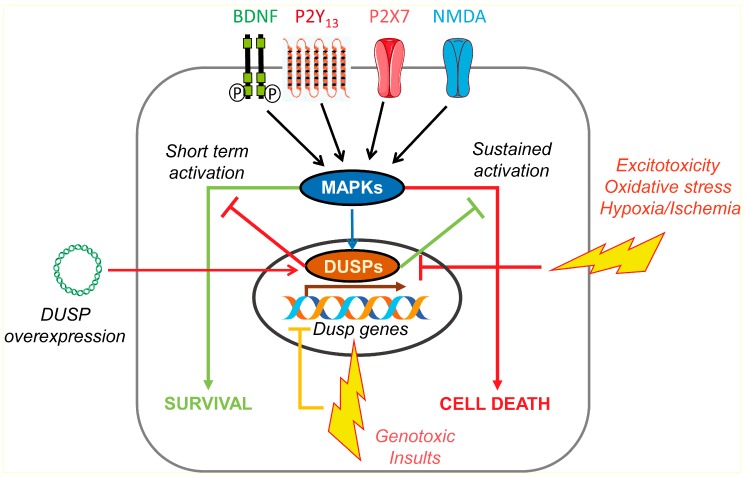
Scheme representing the neuroprotective role of some neurotrophins and nucleotide receptors through the regulation of MAPK and DUSP activity in neural cells. Tyrosine kinase receptors (BDNF, EGF, NGF and FGF), NMDA, and nucleotide ionotropic P2X7 and metabotropic P2Y_13_ receptors regulate DUSP1, DUSP2, and DUSP6 levels through different mechanisms, involving transcriptional induction, turnover regulation, and protein stabilization. The reestablishment of DUSP activity is essential to avoid the over-activation of MAP kinases and cell death induced by different apoptotic insults in neurons and glial cells. Arrows indicate activation of MAPKs, DUSP expression and cell survival/death. T-bars indicate inhibition of MAPK activation and DUSP expression and activity.

From the above results, it is clear that DUSP activity is required to maintain neuronal survival and homeostatic control of MAPK signaling (Figure 2). However, it is necessary to take into account that the final effect of increasing DUSP activity depends on the cell type and severity of the stimulus and whether the stress is in the acute or delayed phase. Strategies of *DUSP* overexpression can be detrimental if they prevent the pro-survival effect of ERK signaling, as described in SH-SY5Y human neuroblastoma cells challenged with H_2_O_2_. In this model, it is *DUSP1* knockdown that prevents apoptosis and exerts a cytoprotective role [68]. *DUSP6* overexpression can turn toxic to oligodendrocyte cultures submitted to glutamate excitotoxicity when abrogating ERK signaling that is required to elicit a full protective response during AMPA receptor activation. As a consequence of *DUSP6* overexpression, AMPA receptor signaling amplifies and calcium overload contributes to oligodendrocyte cell death. The fact that DUSP6 increases in rat optic nerves exposed to excitotoxicity and in rat optic nerves of patients with multiple sclerosis suggests that *DUSP6* overexpression might be a risk factor of vulnerability in early stages of this disease [69].

### 4.2. DUSP Regulation in Genotoxic Stress

Altered MAPK signaling also promotes cell death in conditions of DNA damage characterized as genotoxic stress and, again, several mechanisms converge in both nuclear and cytoplasmic compartments to inactivate DUSP proteins. These include direct oxidative phosphatase inactivation, as oxidative damage is also inherent to genotoxic stress, and deficiencies in the *DUSP* transcription machinery. In fact, *DUSP*s are among the genes that become transcriptionally inhibited during exposition to DNA-damaging agents [70]. 

Exposure to the chemotherapeutic agent cisplatin in primary mice cortical neurons promotes genotoxic stress and is enough to induce long-lasting ERK activation that functions as a defense mechanism. In these conditions, the kinetics of ERK dephosphorylation lower and explain how phosphorylated ERK accumulates in response to the basal activity of NMDA and BDNF receptors. Further analysis demonstrates that a decline in *DUSP6* mRNA might be the consequence of transcriptional inhibition upon cisplatin treatment in cortical neurons. In fact, the use of transcription inhibitor actinomycin D not only diminishes *DUSP6* mRNA, but also prevents efficient ERK dephosphorylation. Among the extracellular mediators able to restore *DUSP6* expression, both NMDA and BDNF increase *DUSP6* transcription and contribute to neuroprotection. The neuroprotective effect of BDNF stimulation during cisplatin treatment depends on the ability to promote ERK pro-survival signaling (Figure 2) [71,72]. 

*DUSP6* mRNA and protein levels also decrease during cisplatin treatment and correlate with ERK over-activation in cerebellar-cultured neurons. Although BDNF and growth factors represent strong signals in this neuronal model, extracellular nucleotides arise as significant regulators of DUSP proteins. The stimulation of several nucleotide receptors results in trophic behavior and the sharing of analogous mechanisms used to modulate signaling cascades with growth factors. In particular, the ERK-directed phosphatase DUSP6 appears to be a signaling target for the nucleotide receptor P2X7 (P2X7R) in primary cultures of rat cerebellar astrocytes and neurons. P2X7R is an ionotropic nucleotide receptor permeable to calcium and for which relevant functions in neural cells have been described [14,73,74,75,76]. In cerebellar neurons and astrocytes, ERK signaling activated by P2X7R is involved in the preservation of cell survival and differentiation [77,78,79]. With this experience, it was not surprising to find that P2X7R regulates DUSP6, one of the main targets of ERK. As expected, with the P2X7R agonist, 2′(3′)-*O*-(4-Benzoylbenzoyl)adenosine-5′-triphosphate (BzATP), is able to induce *DUSP6* gene transcription in an ERK-dependent way, which accounts for its protective effect in conditions of genotoxic stress induced by cisplatin and UV light (unpublished results) (Figure 2). 

Interestingly, both astrocytes and granule neurons represent good examples of the fine regulation of DUSP6 activity in neural cells. *DUSP6* expression levels varied along with time of incubation with BzATP, exhibiting a biphasic profile. During short incubation periods, the DUSP6 protein rapidly and significantly decreases below basal levels, and then increases until recovery in neurons or beyond basal levels in astrocytes [80]. The first phase of DUSP6 protein loss is due to protein degradation, because it can be prevented by the use of a proteasome inhibitor. At least in astrocytes, it was demonstrated that ERK-mediated phosphorylation of DUSP6 at the Ser-197 residue could contribute to initial DUSP6 protein decline, because this residue is involved in targeting DUSP6 for proteasome degradation [38]. This biphasic mechanism was first described for DUSP6 by Jurek and co-workers [39]. In porcine aortic endothelial cells ectopically expressing the platelet-derived growth factor (PDGF) receptor, PDGFR activation promoted rapid DUSP6 degradation. Nevertheless, in this study, the residues involved in destabilizing the protein were Ser-300 and Ser-174 [39], different to what was found in astrocytes. Therefore, different residues could be susceptible to ERK-mediated phosphorylation for different stimuli and cell contexts.

The ERK-dependent transcription of the *DUSP6* gene is responsible for the recovery phase of DUSP6 protein levels in cerebellar neurons and astrocytes stimulated with the P2X7 agonist and in aortic cells stimulated with PDGF. The reestablishment of DUSP6 protein levels at later times helps to efficiently terminate ERK signaling [15,39]. The epidermal growth factor (EGF) reproduces the same pattern of biphasic regulation of DUSP6 in both cerebellar astrocytes and neurons, suggesting that this represents a common and universal mechanism shared by different mediators in neural and non-neural cells [15]. The physiological meaning of DUSP6 biphasic regulation allows ERK to regulate its own activity, by participating in positive feedforward and negative feedback mechanisms. Initially, DUSP6 down-regulation amplifies ERK-directed activity towards its cytosolic substrates to enlarge pro-survival signaling. This is followed by DUSP6 protein recovery to finish ERK activation and avoid inappropriate long-term activation of pro-apoptotic signaling (Figure 2). These data, together with the vulnerability of neurons and astrocytes to the BCI-specific DUSP1/6 inhibitor, indicate that the fine regulation and preservation of DUSP6 expression and activity is necessary to maintain cell survival, which can be accomplished by P2X7R stimulation.

As far as P2X7R provides a mechanism for reciprocal adaptation between ERK signaling and DUSP6 activity, examples of cross-regulation of MAPKs by DUSPs also occur with other nucleotide receptors [12,13]. In the same models of cerebellar neurons and astrocytes, the ADP-responding nucleotide metabotropic receptor P2Y_13_ (P2Y_13_R) is involved in the regulation of the nuclear and inducible phosphatase, DUSP2 (PAC-1). *DUSP2* was one of the over-represented genes related to protein phosphatase activity that were identified in microarray expression analysis of P2Y_13_R-stimulated neurons. The transcriptional induction of *DUSP2* by P2Y_13_R stimulation requires both PI3K- and ERK-dependent signaling [81]. The above results suggest that a certain level of specialization takes place in the specific DUSP regulated by different extracellular nucleotides and growth factors concurring in the same cellular type. It appears that DUSP6 is preferentially regulated by P2X7 and EGF receptors, while DUSP2 is the target of P2Y_13_ receptors in cerebellar neurons and glial cells (Figure 2) [15].

As well as DUSP6, genotoxic stress challenge in cerebellar neurons also influences the activity of DUSP2. Both DUSP2 mRNA and protein levels decrease concomitantly with DNA damaging agents, UV light, and cisplatin, while ERK and p38 phosphorylation are increased. Interestingly, the preferred MAPK substrate for DUSP2 in cerebellar neurons seems to be p38, as *DUSP2* expression recovery mediated by P2Y_13_R stimulation prevents the accumulation of the p38 phosphorylated form in the nucleus and increases cell survival [81]. Therefore, it is normalized DUSP2 expression that explains the neuroprotection elicited by P2Y_13_R in granule neurons exposed to cytotoxic cisplatin. 

Maintaining DUSP expression and activity is also protective in other neuronal types submitted to apoptotic stimuli. The withdrawal of trophic factors induces *DUSP1* expression in sympathetic neurons [82]. Similarly, in conditions of ER-induced stress, DUSP1 protein stabilization by phosphorylation in Ser-359 maintains survival in cerebellar neurons [83]. Even in non-neural cells exposed to different apoptotic stimuli, *DUSP1* induction is important to counteract apoptotic p38 and JNK signaling in HEK293 cells and in mouse embryo fibroblasts [21,22,84]. All these data together suggest that the induced expression of DUSPs is the mechanism shared by neural and non-neural cells to maintain survival. 

### 4.3. DUSP in Hypoxia and Ischemia

In the neural context, DUSP1 forms part of the endogenous response to brain hypoxic and ischemic injury. Genome-wide gene expression analysis reveals the *DUSP1* gene as one of the survival genes upregulated in the rat hippocampal CA1 region in response to global cerebral ischemia [85]. Additionally, this phosphatase also appears to be involved in ischemic preconditioning in rat retina. *DUSP1* overexpression is essential to prevent the cell apoptotic program activated by JNK and p38 signaling in different cellular layers of the inner retina [86]. *DUSP1* expression also increases the survival of cortical neurons and neuroblastoma cells when submitted to hypoxia-deoxygenation, while *DUSP1* silencing abolishes this protective effect [87]. The neuroprotective role of *DUSP1* overexpression has also been reproduced in vivo in the middle cerebral artery occlusion (MCAo) rodent model of ischemia. Increased levels of the DUSP1 protein are detected in the peri-infarct region after stroke. Through counteracting the deleterious activation of JNK and p38 signaling, *DUSP1* expression prevents the apoptotic program in neurons and limits the inflammatory response in microglia. In agreement, DUSP1 pharmacological inhibition or genetic deletion increases infarct size, exacerbates neurological deficits, and worsens the final outcome, facilitating hemorrhagic transformation [88].

## 5. DUSP in Pain and Inflammation; Role of Cannabinoids

In certain pain paradigms that present pain hypersensitivity, activated MAPKs behave as transducers of signaling cascades involved in the initiation and maintenance of mechanical allodynia and inflammation. ERK and p38 proteins are mainly altered following peripheral nerve injury and mechanical-induced hypersensitivity in different spinal cell populations, especially in neurons and microglia. Furthermore, adaptations at the level of DUSPs also occur and DUSP proteins levels decrease in different pain conditions. As occurred in apoptotic conditions, the restoration of *DUSP* expression can have therapeutic value in the resolution of pain and inflammation. With regards to this, in a model of neuropathic pain, the overexpression of *DUSP1* phosphatase by genetic approaches in the spinal cord of rats is enough to prevent mechanical allodynia. This anti-nociceptive effect is followed by a decrease in pro-inflammatory mediators and lower levels of phosphorylated p38 in the spinal cord [89]. In agreement, the studies performed with genetic *DUSP6* knock-out mice also confirm that *DUSP6* expression is essential to achieve a spontaneous resolution of mechanical allodynia during post-operative pain [90]. The lack of the DUSP6 protein specifically alters the regulation of ERK in mice paw tissue after a surgical wound incision, indicating that also in the periphery, DUSP6 impairment contributes to maintaining post-operative allodynia [91]. 

Alternatively, *DUSP* expression recovery can be achieved by several pharmacological interventions. Among different mediators, endogenous cannabinoids behave as strong regulators of DUSP activity. Cannabinoids work as retrograde messengers that integrate the strength of synaptic inputs and activity to regulate synaptic transmission in the nervous system. When released in response to brain injury, they function to maintain survival and attenuate cell damage through the activation of CB1 and CB2 receptors. According to their neuroprotective role, cannabinoids have been revealed as potent inducers of *DUSP* expression, especially in inflammatory conditions.

Studies carried out in microglial cell models demonstrate that the stimulation of cannabinoid receptors, CB1 and CB2, limits inflammatory responses through the modulation of DUSP activity. In microglial cells treated with lipopolysaccharide (LPS), the CB2 agonist JWH015 increases the expression of both DUSP1 and DUSP6 protein phosphatases. This effect terminates ERK signaling and thereby prevents ERK-mediated migration and TNF-α production, two hallmarks of microglial reactivity [92]. Moreover, in BV-2 murine microglial cells submitted to an LPS inflammatory stimulus, *DUSP1* induction is mediated by the CB1/2 receptor activated by anandamide and WIN 55,212-2 agonists. The increase in DUSP1 protein levels switches off ERK signaling to limit the local immune response. Interestingly, *DUSP1* induction by cannabinoids is achieved through a novel mechanism that involves histone H3 phosphorylation, being independent of ERK signaling [93]. The regulation of *DUSP1* gene expression by histone remodeling also takes place following the arrival of different types of stressful stimuli, such as UV light, hydrogen peroxide, and heat shock in mouse embryo fibroblasts [25]. 

Endocannabinoids can also rescue retinal Müller glia from inflammatory damage induced by LPS. Anandamide and 2-arachidonoylglycerol acting through CB1 and CB2 receptors induce *DUSP1* expression, leading to the subsequent dephosphorylation of MAPKs, prevention of the NF-κB transcription complex, and synthesis of pro-inflammatory cytokines. Through the modulation of MAPK signaling, cannabinoids also increase the expression of tristetraprolin (TTP) in activated Müller cells, which binds and promotes the destabilization of AU-rich pro-inflammatory cytokine mRNAs, contributing to the suppression of cytokine protein levels [94].

More importantly, the effect of cannabinoids in *DUSP* expression is not restricted to cultured isolated cells, but also reproduced in vivo. Studies of peripheral nerve injury show that the intrathecal administration of CB2 agonist JWH015 also increases the expression levels of *DUSP1* and *DUSP6* phosphatases in both neuronal and microglial cells of the spinal cord in a rat nerve transection model of neuropathic pain. It is the restoration of phosphatase activity that re-establishes the physiological levels of pERK and p38 in spinal cord cells and relieves pain [95].

**Figure 3:**
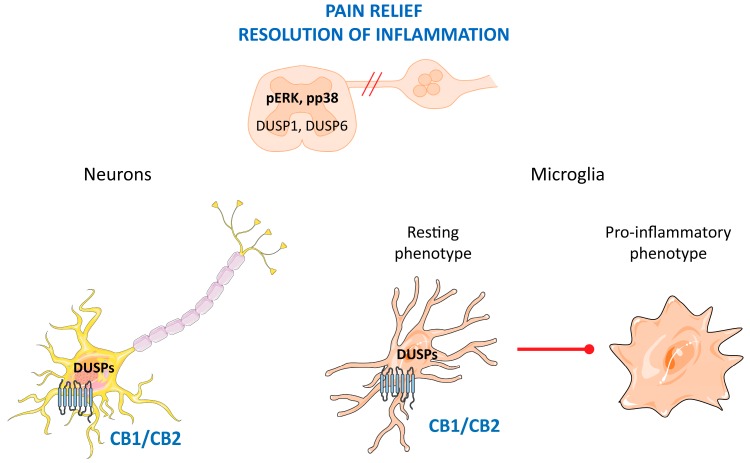
Role of cannabinoid receptors in pain relief and inflammation. CB1 and CB2 receptors increase *DUSP1* and *DUSP6* expression in neurons and microglial cells, which contributes to counteracting the over-activation of ERK and p38 in different pain models. In microglial cells, *DUSP1* expression induction takes place by a novel mechanism involving histone remodeling, which is independent of MAPK activation. DUSP activity prevents the microglia activation and the release of pro-inflammatory mediators.

All these data together point out the crucial role of preserving *DUSP* expression in spinal cell populations to prevent alterations in MAPK activity during the establishment of pain. Therefore, DUSP phosphatases also behave as targets for the development of novel analgesic therapies and a new approach could be based on the activation of cannabinoid receptors in the spinal cord (Figure 3).

## 6. DUSP in Brain Diseases

### 6.1. DUSP in Neurodegenerative Diseases

Concerning the important implications of DUSPs in neuroprotection, differentiation, and inflammation, it is not surprising to find that they become dysregulated in neurodegenerative diseases. Aberrant MAPK signaling represents a central feature of cell death pathways, such as oxidative stress, endoplasmic reticulum stress, mitochondrial dysfunction, and alterations in cellular proteostasis. During neurodegenerative events, the phosphorylation state of direct and indirect MAPKs substrates becomes altered and contributes to the assembly and stabilization of toxic protein aggregates characteristic of neurodegenerative diseases [96,97,98,99,100]. It is worth noting that among MAPKs, ERK signaling dysregulation can have a major impact on neurodegeneration, because it is essential for neurotransmission, synaptic plasticity, neuronal differentiation, and survival.

A misbalance between ERK activity and *DUSP* expression appears to be clear in Alzheimer′s disease (AD). Downregulation of *DUSP6* is observed in post-mortem samples of AD patients, reaching a 50% decrease in frontal cortex lysates [101]. Taking into account that lower levels of *DUSP6* transcripts correlate with the over-expression of miR-125b observed in AD cortices, this study concludes that DUSP6 is a direct target of miR-125b and its dysregulation contributes to facilitating tau-mediated cytotoxicity. Indeed, when miR-125b is overexpressed in hippocampal neurons or directly injected into a mouse hippocampus, it reproduces several hallmarks of AD, such as tau hyperphosphorylation, sustained ERK activation, and DUSP6 decrease [101]. The potential neuroprotective role of *DUSP6* in AD is suggested by the results obtained in the C17.2 neural stem cell line, in which *DUSP6* overexpression protects against amyloid peptide fragment (Ab 31–35) toxicity and restores normal ERK signaling [102].

Concerning the relevant role of DUSP1 in the regulation of synaptic plasticity and neuronal morphology, impaired physiology of DUSP1 is also evident in Alzheimer’s disease (AD). DUSP1 levels are also diminished in cortical tissues obtained from AD patients and correlate with tau pathology, cognitive decline, and high blood levels of glucocorticoids; the latter represents a risk factor for AD. In models of glucocorticoid resistance, *DUSP1* downregulation can be explained by the deactivation of BDNF signaling, taking into account that *DUSP1* is a transcriptional target of BDNF and glucocorticoids [103,104]. The delivery of minigene constructs overexpressing *DUSP1* in a mouse cortex and in primary cortical neurons has the ability to prevent tau hyperphosphorylation and other neurochemical deficits caused by deficient glucocorticoid and BDNF-dependent signaling [105].

Similarly, downregulation in *DUSP1* transcripts also occurs in the cortex and striatum of mice models of Huntington’s disease (HD) and post-mortem samples of HD patients [106,107]. In addition, the loss of the DUSP1 protein and mRNA in mouse cortical and striatal neurons submitted to polyglutamine-expanded huntingtin toxicity correlates with a lack of dephosphorylating activity towards p38 and JNK. It is worth mentioning that neuroprotection is achieved when *DUSP1* expression recovers, both in cultured neurons and in rat striatum. The protective effect clearly involves ameliorating pro-apoptotic p38 and JNK signaling and preserving pro-survival ERK-dependent activity [107]. In line with this, the histone deacetylase inhibitor, sodium butyrate, is neuroprotective in the R6/2 transgenic model of HD and mediates the upregulation of *DUSP1* expression [108].

DUSP1 and DUSP6 phosphatases also behave as molecular markers of the progression and prognosis of Parkinson’s disease (PD). In rat primary dopaminergic neurons challenged with 6-OHDA, a neurotoxin commonly used to model PD, 6-OHDA-induced cytotoxicity is dependent on a DUSP1 decrease and concomitant over-activation of p38 signaling. The protective effect is achieved by the overexpression of *DUSP1*. Moreover, overexpression also restores normal neuronal morphology and promotes neurite outgrowth and a normal degree of neuronal branching and complexity [51,109]. Transcriptome studies also reveal that *DUSP1* and *DUSP6* transcripts are among the most significantly upregulated genes in a mouse hemiparkinsonism model submitted to chronic L-Dopa treatment. *DUSP* gene expression induction can then function as a defense mechanism to overcome neural damage [110,111]. These results suggest that the search for *DUSP1* and *DUSP6* expression inducers can be a good therapeutic alternative in the treatment of neurodegenerative events.

### 6.2. DUSP in Neurological Disorders

In a different way to the specific loss of *DUSP* expression during apoptotic and neurodegenerative events, the opposite seems to occur in the context of neurological disorders, which seems to correlate with an increase in DUSP proteins. Moreover, the relationship between DUSPs and neurological disorders is supported by a strong genetic basis. The appearance of some *DUSP6* SNPs or genetic variants constitutes risk factors, among others, in some psychiatric diseases, such as bipolar disorder (BD). The *DUSP6* gene is located on chromosome region 12q22-q23 identified as bipolar disorder susceptibility loci. Wide genetic association studies reported a positive association between some missense mutations of *DUSP6* and BD, especially in females [112,113,114]. These missense mutations progress with a gain of function and weaken intracellular ERK signaling in response to lithium treatment. Therefore, changes in DUSP6 activity can have a strong impact on the therapeutic efficacy of the conventional BD treatment regimen [114]. In accordance with these data, hypoactivity in the ERK signaling cascade is reported in postmortem brains of BD patients [115,116].

With respect to DUSP1, this phosphatase appears to be more related to the pathophysiology of depressive behaviour and DUSP1 levels increase in the hippocampus of depressive patients and stressed rats [117]. The ERK-pathway is the most affected in depression and reflects on deficits in ERK-directed gene expression of key mediators of mood-relating function, such as BDNF and vascular endothelial growth factor (VEGF) [118]. Interestingly, the local overexpression of *DUSP1* in the hippocampus reproduces depressive symptoms in mice. In agreement, lack of the DUSP1 protein protects from depressive behaviour, indicating that inhibition of DUSP1 activity can be a promising therapeutic approach for the treatment of depression. In line with this, selective serotonin reuptake inhibitors (SSRIs) currently used as antidepressants are able to reduce DUSP1 levels [117,119]. The first attempt to assay DUSP1 inhibitors in the treatment of depression focused on Sanguinarine, a natural plant-derived alkaloid with the capacity to selectively inhibit DUSP1 [120]. The intracerebral infusion of Sanguinarine at the ventrolateral orbital cortex relieves the depressive behaviour of rats to a similar extent as that obtained with the systemic administration of the classical antidepressant fluoxetine. The specificity of Sanguinarine at the molecular level is demonstrated because it decreases DUSP1 cortical levels and promotes a concomitant increase in pERK [121].

### 6.3. DUSPs in Brain Tumors

Most of the dual phosphatases implicated in brain cancers have been identified in glioblastoma (GB), the most common, malignant, and lethal primary intracranial tumor in adults [122]. GB is characterized by its intrinsic aggressiveness and poor prognosis, showing a high heterogeneity at clinical, morphological, molecular, and cellular levels. Currently, GB is treated with surgical resection, radiotherapy, and daily chemotherapy with temozolomide, an oral alkylating agent that triggers tumor cells death by inducing DNA damage, but frequently, the tumor recurs. During the last decade, DUSPs have been proposed as relevant regulators of GB behavior, being involved in either tumor-suppressor or tumor-promoter mechanisms, depending on the phosphatase subtype and the specific context [123,124]. Analysis of a broad range of human GB tumors revealed that *DUSP*1 and *DUSP6* exhibit the highest expression level compared to other *DUSPs* [125]. This is noteworthy considering the different but complementary subcellular distribution and MAPK specificity of *DUSP1* and *DUSP6*. Different groups have reported that DUSP1 is upregulated in response to low-oxygen conditions or chemotherapeutic exposure. Thus, hypoxia [125,126] and DNA-damaging agents, such as camptothecin [125] and temozolomide [127], induce *DUSP1* expression in GB cells. Moreover, both dexamethasone and rogisiglitazone reduce cell invasiveness through *DUSP1* induction in human malignant GB cells [128,129]. Recently, Arrizabalaga et al. (2017) studied DUSP1 levels in two sets of independent glioma human samples, revealing that a clinically high expression of *DUSP1* positively correlates with increased GB patient overall survival. This study also showed that the overexpression of DUSP1 in glioma cell lines significantly reduces cellular growth in vitro and in vivo, mainly by suppressing the proliferation and self-renewal ability of the glioma stem cells niche [127]. On the contrary, DUSP6 has been reported as a tumor-promoting factor in human GB [130]. Thus, *DUSP6* overexpression in primary and long-term cultures of human GB enhances tumor growth and increases resistance to cisplatin-mediated cell death in vitro and in vivo [131].

## 7. Concluding Remarks

The present work covers the data regarding the regulation of DUSP expression and activity by several extracellular messengers of physiological relevance in neural cells. DUSP regulatory mechanisms in cell populations of the nervous system match the modes of DUSP regulation found in peripheral tissues. Additionally, the identification of neurotrophins, cannabinoids, and nucleotides as new players in DUSP regulation place them in a position to intervene in processes of brain pathology.

**Figure 4:**
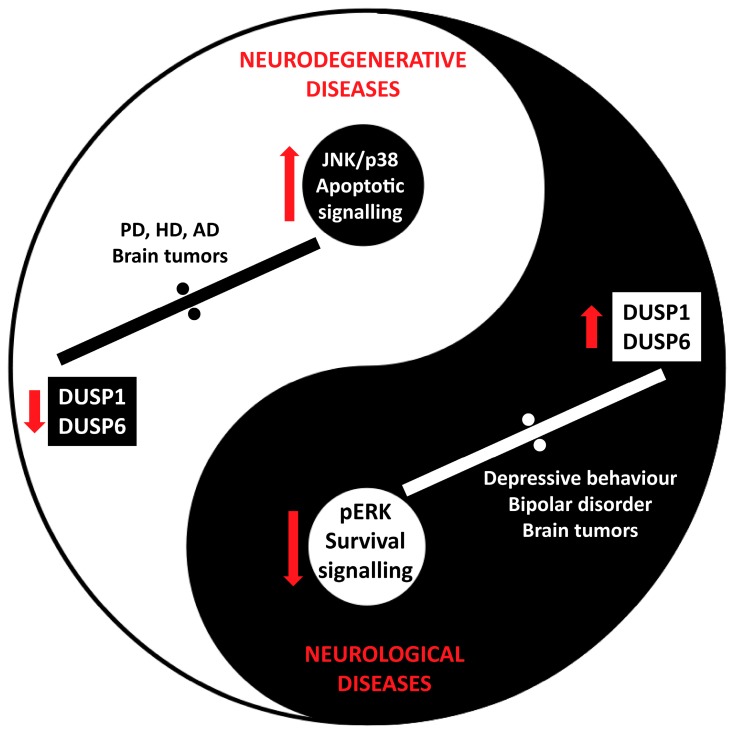
The yin and yang of DUSP activity in brain diseases. This figure summarizes the current available data about DUSP activity in brain diseases, but they only represent the tip of the iceberg. Whereas the increase in *DUSP* expression can be beneficial for neurodegenerative diseases, it is detrimental for neurological disorders. The restoring of the appropriate levels of DUSP activity will be determined by the injury type and the spatiotemporal context.

It is important to remark that increasing *DUSP* expression or restoring DUSP levels is a valid approach to obtain neuroprotection in brain diseases. In agreement, the increased expression of phosphatases, mainly DUSP1 and DUSP6, exhibits a pro-survival effect against the arrival of stressful stimuli that compromise cell survival, such as H_2_O_2_, UV, cisplatin, hypoxic insults, and during excitotoxicity and ischemic injury. In studies performed in primary cultures of neurons and astrocytes, neuroprotection is achieved by restoring accurate *DUSP* expression levels and activity to counteract the pro-apoptotic activation of p38- and JNK-mediated signaling. These approaches are specifically important in vivo when neurodegeneration has been fully installed in brain tissue and the adaptive mechanisms are overwhelmed. It is not surprising to find striking changes in the expression levels and activity of these phosphatases in neurodegenerative diseases, as occurs with the downregulation of *DUSP1* and *DUSP6* in AD and HD. In these conditions, sustained changes in MAPKs accumulate over time and are responsible for several of the toxic events related to neurodegeneration, such as apoptosis, protein aggregation, and changes in cytoskeletal dynamics. However, it is necessary to take into account that the overexpression of *DUSPs* can also be detrimental if it prevents pro-survival ERK signaling, as demonstrated in some in vitro models, in which it is DUSP inhibition that prevents cell death. In addition, a balance between mitogenic and differentiating signals is required to ensure the proper processes of neuronal differentiation, arborization, and synaptic connectivity during development and DUSP activity needs to be accurately regulated in the precise stage of developing tissue. Other examples of the deleterious effect of increased DUSP activity are found in neurological disorders. DUSP6 and DUSP1 gains of function behave as markers of the progression and prognosis of bipolar disorder and depressive behavior, respectively, and they are responsible for deficient ERK signaling in these pathologies (Figure 4).

Concerning brain tumors, the great variability found in DUSP activity makes it difficult to apply a common therapeutic strategy based on modifying DUSP alterations. In glioblastomas, DUSPs can behave either as tumor suppressors or promoters, depending on the stage and progression of a specific cancer type. Much more needs to be addressed regarding brain tumors to establish a therapeutic approach based on inhibitors or inductors of DUSP. All these data together indicate that the actuation at the level of augmenting or inhibiting DUSP activity will depend on environmental changes and different pathological contexts (Figure 4).

From the perspective that the recovery of DUSP levels and activity is a valid approach to obtain neuroprotection during brain damage, it is important to better understand to what extent DUSP activity is modulated through specific neural mediators. In this role, neurotrophins and cannabinoids represent the most potent inducers of DUSP expression and stabilization in neural cells. The relevance of endogenous cannabinoids in the regulation of DUSP activity is based on their availability to use cannabinoid agonists in vivo for the resolution of pain and inflammation. Activation of BDNF signaling in the brain might ensure proper neuronal cytoarchitecture and cell survival through the regulation of DUSP1. At the same time, restoring BDNF signaling is imperative in depressive disorders. Therefore, different approaches to potentiate BDNF functions would be of great interest in brain pathology.

DUSPs also represent new elements in the nucleotide signaling network. In cerebellar cells, P2X7 and P2Y_13_ nucleotide receptors specifically regulate DUSP6 and DUSP2, respectively. Additionally, nucleotides can integrate DUSP regulation elicited by other mediators. P2X7 and BDNF receptors interact at the level of glycogen synthase kinase 3- (GSK3)-coupled signaling in cerebellar neurons and amplify the survival responses elicited by BNDF [132]. It is tempting to speculate that such interactions can also take place at the level of DUSP regulation and could represent an alternative approach to potentiate and amplify BDNF-mediated signaling. The same could be true for nucleotide and cannabinoid receptors. Overall, DUSPs represent important convergent points of signaling networks that encompass the signaling cascades activated by different mediators in the nervous system.

## Abbreviations

AD	Alzheimer′s disease
BD	Bipolar disorder
BDNF	Brain-derived neurotrophic factor
BzATP	2′(3′)-*O*-(4-Benzoylbenzoyl)adenosine 5′-triphosphate
DUSP	Dual-specificity protein phosphatase
EGF	Epidermal growth factor
ERK	Extracellular signal regulated protein kinase
ETS	E twenty-six
GSK3	Glycogen synthase kinase 3
H_2_O_2_	Hydrogen peroxide
HD	Huntington′s disease
JNKs	c-Jun N-terminal kinase
MAPK	Mitogen-activated protein kinase
MEK	Mitogen extracellular activated kinase
NGF	Nerve growth factor
PD	Parkinson′s disease
PI3K	Phosphatidylinositol 3-kinase
PKA	Protein kinase A
PKC	Protein kinase C
PTP	Protein tyrosine phosphatase
TTP	Tristetraprolin
